# *Vis Medicatrix naturae*: does nature "minister to the mind"?

**DOI:** 10.1186/1751-0759-6-11

**Published:** 2012-04-03

**Authors:** Alan C Logan, Eva M Selhub

**Affiliations:** 1 Genuine Health, 775 East Blithedale Avenue, Suite 364, Mill Valley, CA 94941, USA; 2 Harvard Medical School, Massachusetts General Hospital, 40 Crescent St., Suite 201, Waltham, MA 02453, USA

## Abstract

The healing power of nature, *vis medicatrix naturae*, has traditionally been defined as an internal healing response designed to restore health. Almost a century ago, famed biologist Sir John Arthur Thomson provided an additional interpretation of the word nature within the context of *vis medicatrix*, defining it instead as the natural, non-built external environment. He maintained that the healing power of nature is also that associated with mindful contact with the animate and inanimate natural portions of the outdoor environment. A century on, excessive screen-based media consumption, so-called screen time, may be a driving force in masking awareness of the potential benefits of nature. With global environmental concerns, rapid urban expansion, and mental health disorders at crisis levels, diminished nature contact may not be without consequence to the health of the individual and the planet itself. In the context of emerging research, we will re-examine Sir J. Arthur Thomson's contention that the healing power of the nature-based environment - green space, forests and parks in particular - extends into the realm of mental health and vitality.

## 

"*What then do I mean tonight by the healing power of nature? I mean to refer to the way in which Nature ministers to our minds, all more or less diseased by the rush and racket of civilization, and helps to steady and enrich our lives. My first point is that there are deeply-rooted, old established, far-reaching relations between Man and Nature which we cannot ignore without loss... there would be less "psychopathology of everyday life" if we kept up our acquaintance... we have put ourselves beyond a very potent vis medicatrix if we cease to be able to wonder at the at the grandeur of the star-strewn sky, the mystery of the mountains, the sea eternally new, the way of the eagle in the air, the meanest flower that blows, the look in a dog's eye*." [1].

Professor J. Arthur Thomson - "*Vis Medicatrix Naturae*" - Keynote Address at the Annual Meeting of the British Medical Association, 1914.

The healing power of nature - *vis medicatrix naturae *- is an ancient medical principle that includes reference to the innate ability of the body to heal itself. While acknowledging that *vis medicatrix naturae *can be influenced by anything from physician bedside manner to belief in placebo, medical scholars have typically defined it as an internal healing response designed to repair and rebuild [2]. Consider, for example, the healing of a fracture; "naturae" in the contemporary context is what we now recognize as the production of immune chemicals and the initiation of enzymatic reactions, a proper balance of pro- and anti-inflammatory cytokines, osteoblast and osteoclast activity etc., in the remodeling of bones. However, a century ago biologist Sir John Arthur Thomson provided an additional interpretation of the word nature within the context of *vis medicatrix*, defining it instead as the natural, non-built physical environment in which humans live their lives - i.e. that the healing power of nature is also that associated with mindful immersion in and contact with the animate and inanimate natural portions of our external world [1]. In our review we will re-examine the contentions of Sir J. Arthur Thomson, and in particular his suggestion that the healing power of the nature-based environment extends into the realm of mental health.

## Screen time and displacement of green time

Some researchers have suggested that a significant portion of modern children and adults may be experiencing suboptimal levels of exposure to green space and time spent in natural settings [3]. Typically nature or natural settings are broadly defined in this context as inclusive of outdoor areas rich in vegetation and non-human animal life, including forests, urban parks, waterside areas and relatively untouched wilderness regions. Several recent studies have suggested that the expansion of screen-based entertainment (televisions, computers, video games, Smartphones) has contributed, at least in part, to the downward trend in nature-based recreation over the last two decades. While this is obviously difficult to prove, the declining visits to national and state parks, historic sites and wilderness areas - down in many locations between 25-50% - have certainly occurred in tandem with massive increases in non-academic and non-occupational daily screen time and screen-based media consumption [4-8]. Most of the research in the area of the health-related consequences of excessive screen time has focused on implications related to obesity, cognitive performance, anxiety and depression. The results of recent prospective studies are now reporting that the accumulation of screen time is a risk factor for, and not a mere consequence of, mental health disorders [9-11]. Perceptions of cyber-based information overload are predictive of more frequent and more severe health problems [12]. Screen time, however, cannot be viewed in isolation, it can be a surrogate marker for lack of physical activity or less time spent in meaningful social interaction with a pronounced health payoff. Those experiencing high levels of cyber-based information overload are much less likely to engage in contemplative activities [12]. It remains unknown to what extent the loss of green time - time spent outdoors in nature, or at the very least, a view to nature - is itself a risk factor for mental health disorders and cognitive difficulties. Put another way, an unanswered question is whether or not the loss of contact with nature, its displacement by the screen, removes a layer of psychological resiliency.

## Nature, stress physiology and brain imaging

Over 30 years have passed since scientist Roger S. Ulrich first began to examine some of the psycho-physiological changes induced by vegetation-rich scenes of nature (relative to urban scenes). His initial studies found that immediately subsequent to a required one-hour course examination, undergraduate students who viewed photographic scenes of nature (vs. urban built scenes) had a rapid improvement in positive mental outlook and a decline in reported fear and arousal [13]. These subjective reports were subsequently corroborated in separate work involving objective markers of stress physiology including electromyography (EMG), skin conductance (SC) and pulse transit time (PTT) [14]. Specifically, after viewing a stressful video on workplace accidents ("*It didn't have to happen*" - a video previously confirmed to elevate a stress response), participants subsequently viewed images of nature scenes or an urban built environment for 10 minutes. The physiological markers (EMG, SC, PTT) showed a consistent pattern of rapid and more complete recovery from stress/arousal upon exposure of vegetation-rich nature scenes. Ulrich was the first to use electroencephalograph (EEG) apparatus to evaluate brain wave activity while otherwise healthy adults viewed photographic scenes of nature vs. urban built scenes [15]. The results confirmed higher alpha wave activity when viewing scenes of vegetation-rich (and aesthetically unspectacular) nature, indicative of a state of relaxed wakefulness and lowered anxiety.

The original work of Ulrich has been validated to some extent by various international investigators. Nature scenes - steams, valleys, river terraces, orchids, forests, farms and bodies of water - have been shown to positively influence the same objective markers of EEG (higher alpha wave activity), EMG (decreased muscle tension) and skin conductance (decreased autonomic arousal) [16-19]. Lower levels of the stress hormone cortisol have been reported in adults subsequent to performing the same mental activities in a garden setting vs. an indoor classroom [20]. In a Japanese investigation, researchers examined physiological stress markers in 119 adults who transplanted non-flowering plants from one pot to another. Compared to adults who simply filled pots with soil, the individuals working hands-on with the plants had higher EEG alpha wave activity, decreased muscular tension as measured by EMG, as well as subjective reductions in fatigue [21].

A variety of separate Japanese studies under the umbrella terms "shinrin-yoku" (which translates as "taking in the forest air", or "forest bathing") and "forest medicine" have shown that spending time walking or contemplating in a forest setting is associated with lower cortisol, lower blood pressure, pulse rate, and increased heart rate variability. Collectively these studies have involved over 1000 subjects in studies centered in some 2 dozen different forests, and in many cases there was a control or cross-over group engaged in the same activity (physical activity and/or contemplation) within an urban built environment [22,23]. Evaluation with near-infrared time-resolved spectroscopy (NITRS), a device which measures oxygen use in the brain via the reflection of near infrared light from red blood cells, reveals that 20 minutes of contemplation in a forest setting (vs. urban control) altered cerebral blood flow in a manner indicative of a state of relaxation [24]. The shift in stress physiology, lowered stress hormones in particular, has also been proposed to explain the improvement in immune functioning of subjects involved in various forest medicine studies. Compared to time spent in urban built environments, visits to forest settings have been shown to improve natural killer cell activity and the production of anti-cancer proteins [25].

The consistent preference for natural scenic views over urban streets is well documented, indeed the preferences for nature scenes are apparent even when they are presented for a mere 1/100^th ^of a second [26,27]. To add to the weight of the EEG and NITRS studies, Korean researchers recently utilized functional magnetic resonance imaging (fMRI) to investigate brain activation patterns while viewing nature [28-30]. In a series of studies, the researchers evaluated brain activity while participants viewed a set of either rural (mountains, forests) or urban built scenes for 2 minutes each, followed by a 30 second rest. To minimize the influence of intrusive thoughts and a wandering mind, every 1.5 seconds a new photo was shown. The urban scenes showed pronounced activity in the amygdala, a region that typically shows enhanced activity in response to aversive stimuli. Hyperactivity of this area has been linked to impulsivity and anxiety, while shifts from negative affect to positive mental outlook are associated with a decrease in amygdala activity. Moreover, chronic stress and the stress hormone cortisol itself may promote amygdala activity, and a consistently overactive amygdala may enhance memorization for negative vs. neutral stimuli, short-circuiting the areas that would otherwise dampen amygdala activity [31,32]. Recently it was reported that otherwise healthy urbanites (vs. rural residents) have enhanced activity in the amygdala while performing challenging cognitive tasks under conditions of perceived social stress [33].

In contrast, the Korean fMRI studies showed that nature scenes produced a pronounced activity in the anterior cingulate and the insula - increased activity in both of these areas is associated with heightened empathy and altruistic motivation [34]. This is an interesting finding when considering that the mere visualization of being in a natural setting (vs. urban center) is associated with experimental altruism in young adults [35]. Meanwhile, greater activity in the anterior cingulate is associated with emotional stability and a positive mental outlook [36], and activity in the insula is associated with love. For example, when individuals are shown photographs of loved ones while in an MRI scanner, insula activity has been shown to increase [37]. Urban scenes did not influence activity in the anterior cingulate or the insula.

## Nature and cognition

In recent years there has been some scientific support to the notion that viewing scenes of nature or engaging in activities within natural settings is favorable to cognitive restoration [38-41]. Objective measurements using an Eye Position Detector System (EPDS) have shown that eye fixations, indicative of the amount of attention engaged when viewing a scene, are significantly lower while viewing highly fascinating nature scenes vs. built urban settings [42]. This suggests that natural settings are less likely to place a burden on the inhibitory pathways in the brain - i.e. in nature there is less energy expended in efforts to filter out non-pertinent stimuli. For example, after researchers induced mental fatigue in subjects via a cognitively demanding task, half of the group then viewed images that had been independently reported to be high in cognitive restoration potential (forests, water views, mountains, ocean side etc). The other half of the mentally fatigued group viewed low restoration pictures such as city streets with multiple cars, industrial zones, housing developments, factories etc. After viewing some 25 photographs of either high or low restorative potential for about 6 minutes, the subjects repeated the same cognitively demanding task for another 5 minutes. Upon repeat testing, the group who viewed the restorative nature scenes had enhanced accuracy in target detection, faster reaction time, and a higher number of correct responses to challenge vs. those viewing urban scenes [43]. The same research group has recently replicated the findings of improved reaction time (after induced mental fatigue and re-challenge with cognitive test) after viewing nature scenes rated high in fascination. They also reported overall better memory recall after viewing scenes of nature vs. built urban scenes [44]. In separate work, researchers induced mental fatigue with a series of challenging brain games designed to place demands on sustained attention. Immediately following a 35 minute period of intense cognitive effort, the subjects either took a walk (for a little less than an hour) in a vegetation-rich park or on city streets. After the walk, the cognitive tests were repeated, the results showing a significant performance difference in favor of those who had spent time in nature. An important finding of the study was that the cognitive restoration was occurring without changes in mood state *per se *in these otherwise healthy adults [45]. In other words, we cannot assume the cognitive gains provided by nature are simply an artifact of a more positive outlook. Recently, Korean researchers set up an experiment to evaluate the cognitive effects of a walk through a pine forest vs. downtown streets. In a cross-over design, the subjects completed cognitive and mood tests before and after a 50 minute urban or forest walk. The results showed the expected elevations in mood among the forest vs. built urban walkers; however, they also showed that only after the forest walks did participants show significant improvements in post-walk cognition [46].

Furthermore, a European study combing aerial photography and standardized cognitive assessments showed that children (aged 4-6) attending schools where play areas had more trees, shrubs and hilly terrain were least likely to present with behaviors of inattention [47]. This finding was not associated with socio-economic status of the children. In a study involving 101 public high schools in Michigan, classroom and primary cafeteria views were scaled for the degree and types of nature (i.e. how much green and the type of green - trees, shrubs, cut grass, athletic fields etc).. Even after controlling for socio-economic factors, class size, age of the school facilities and other factors, the results showed that classroom and cafeteria views to green vegetation were significant factors in academic performance on standardized tests. Moreover, views to trees and shrubs were associated with higher graduation rates and future plans for attendance at 4-year university programs. Unlike trees and shrubs, a view to a well kept lawn was not associated with academic performance [48].

Based on the successful results using photographic images of nature, it might seem safe to presume that a virtual nature view (a wall mounted plasma TV displaying a window view to nature) might also afford cognitive benefit. Researchers examined this question in a study involving 90 young adults; subjects completed of a series of complex cognitive tasks for 30 minutes at a workstation either close to an actual window view to a nature scene, or close to a wall-mounted high-definition flat screen TV of similar size to the window with the scene depicting that of the actual nature view, or, in the third group, simply facing a blank wall. Lighting level was kept constant for each group. Each participant remained at the workstation for a 5 minute waiting period before and after the cognitive tasks, during which they could freely gaze. The actual window view held the participants attention longer than did the same view depicted on a plasma screen, and physiological markers of stress showed greatest recovery in the group who viewed the actual nature scene outside the window vs. either the plasma TV set or the blank wall. The plasma TV was better than a blank wall, but not as good as a view of nature as impeded only by a thin pane of glass [49].

Researchers from the USA first reported almost a decade ago that green outdoor activities may be associated with symptom reduction of ADHD (attention-deficit hyperactivity disorder) vs. the same activities conducted in built environments. Initial parental surveys suggested that the greenness of play areas was associated with milder symptoms of attention deficit, and that windowless indoor play areas were associated with more severe symptoms [50]. Following up with a larger study, the researchers used data from 452 parents of children formally diagnosed with ADHD and examined the setting of some 50 different activities (from reading to playing sports) to determine if there were differences in attention. Regardless of age, the presence or absence of hyperactivity in the child, economic status, geographic location with the USA, and rural or urban residence, green outdoor activities were associated with symptom reduction [51]. More recently, investigators have performed cognitive testing of attention in children with ADHD after time spent in natural vs. built environments. In a European study, researchers conducted a test of concentration after children had engaged in a period of light to moderate physical activity in a natural wooded area or a built town area. The results showed that performance on concentration tasks were higher in the wooded environment [52]. In a separate study, children with diagnosed ADD completed a series of challenging puzzles designed to tax cognitive attention, after which they walked in one of three different environments for 20 minutes. One group walked through a vegetation-rich urban park, another in a downtown built area, and the third in a residential area clustered with houses. The child was subsequently driven back to a quiet indoor setting for a series of cognitive evaluations of attention and executive functioning. The results were clearly in favor of the urban park as a means of cognitive restoration [53].

Studies have also shown that simulated drives through natural settings (forest roads) appear to be less taxing to the autonomic nervous system vs. simulated drives through urban settings [54]. In experimental research, the presence of equal levels of traffic noise was presented to adult volunteers with two different visual environments, one rich in vegetation and the other an urban built city scene. The results showed significantly less psychological distress and amplified signs of relaxation via EEG assessment when the noise was presented with views to green vegetation. In separate work involving 106 adults, researchers showed that the amount of vegetation along a highway may help mediate driver frustration. In this case the volunteers were cognitively fatigued with mental challenges, after which they proceeded on a simulated drive where the modified variable was roadside vegetation. The participants had a much higher threshold for frustration tolerance after simulated driving on roadways with more vegetation in sight. Furthermore, the researchers had the drivers work at a complex cognitive task after the differing simulated drives. Participants who drove on the high vegetation parkways were less likely to give up on the post-drive mental challenge, working at it for a significantly longer period than those who had driven in the built areas [55].

A variety of studies have also shown that indoor vegetation can also make a difference in cognitive performance. For example, researchers compared a no vegetation room to one manipulated by the addition of four plants (two small flowering plants on a window ledge, a 1-foot tall green plant on the desk and a 4-foot tall floor plant). The participants were asked to perform memory recall and complex proof-reading exercises, and those operating in the room with potted plants showed improved performance between baseline and an evaluation 10 minutes later [56]. Furthermore, Japanese researchers manipulated a small office room and reported that the presence of a 4-foot tall corn plant improved mood and performance scores among women on a task designed to evaluate creativity [57]. Recently, researchers from Australia have reported that indoor plants placed in a classroom may influence academic scores among younger students [58]. Specifically, the researchers placed just 3 plants in half of classrooms belonging to middle-school students of 3 different Brisbane, Australia school districts. There were over 350 students involved, all of whom completed standardized academic tests prior to plant installation and again six weeks following the placement of plants in select rooms. Researchers reported significant improvements in mathematics, spelling and science among students drawn from classrooms where the plants had been placed. The results await scientific peer-review and formal publication.

## In vivo psychotherapy

It has been postulated that in vivo (Latin for "within the living") counseling may, in select cases, offer some advantages over traditional office-based therapy. In the past, wilderness and other natural settings have been described as helpful for group psychotherapy, particularly when incorporated into so-called camp or wilderness therapy [59,60]. While there has been little in the way of proper scientific evaluation of these broad claims of success, a recent study does suggest merit to nature-based psychotherapy. In a study involving 63 patients with moderate to severe depression, participants were assigned to once-weekly cognitive-behavioral therapy in either a hospital setting or a forest setting (arboretum), while a third group acted as a control and were treated using standard outpatient care in the community. The overall depressive symptoms were reduced most significantly in the forest group, and the odds of complete remission were relatively high - 20-30% higher than that typically observed from medication alone. Moreover, the forest therapy group had more pronounced reductions in physiological markers of stress, including lower levels of the stress hormone cortisol and improvements in heart rate variability, a marker of adequate circulatory system response to stress. The researchers conclude that the settings wherein psychotherapy is conducted are not merely 'places', rather they can become part of the therapy itself [61]. Although much more research is required, the results certainly lend credence to *vis medicatrix naturae *as interpreted by Sir J. Arthur Thomson, and they support the claims of ecopsychologists currently conducting psychotherapy in natural settings [62].

## Nature, mood and mortality

Epidemiological investigations provide further support to the subjective and objective findings indicating that nature is a stress buffer of sorts [63,64]. Neighborhood greenness within urban geography is associated with individual life satisfaction and perceived satisfaction with the neighborhood itself [65]. Among over 4,500 adults, those living within a 3 km radius containing a high amount of green space (as measured by National Land Cover Classification Database) were less likely to experience negative health impacts of stress. Among those who had experienced recent life stressors (major losses, financial problems, relationship problems, legal issues etc), having a more dense green space within 3 km radius was associated with fewer health complaints vs. those with a low amount of green space [66]. A separate study involving over 11,000 adults from Denmark showed that living more than 1 km away from green space (forests, parks, beaches, lakes) were 42 percent more likely to report high stress and had the worst scores on evaluations of general health, vitality, mental health and bodily pain [67]. In addition, after examining the medical records of 195 family physicians, Dutch researchers reported that the annual prevalence rate of 15 of the top 24 disease states were lowest among those with the highest green space within a 1 km radius from home. A mere 10% increase in green space vs. group average was associated with resiliency against chronic disease. Those with only 10% green space within 1 km had a 25% greater risk of depression and a 30% greater risk of anxiety disorders vs. those with the highest area of green space near the home [68].

If neighborhood greenness can positively influence mental outlook, stress physiology, and human immune system defense, it would seem reasonable to presume that neighborhood green space might be associated with lower mortality. Japanese researchers recently compared data on the percentage of forest coverage in all Prefectures and national cancer mortality rates provided by the Ministry of Health. After controlling for smoking and socioeconomic factors, there was a significant association between higher forest coverage within Prefectures and lower rates of various cancers - lung, breast, uterine, prostate, kidney and colon cancers [69]. In a study involving the residents of Shanghai, China, researchers reported that a higher proportion of neighborhood parks, gardens and green areas were associated with a reduced risk of mortality [70]. In the USA, researchers examined 5 years worth of data on stroke mortality and found that geographic green space (as measured via satellite technology) offered significant protection, while areas low in green space were associated with a significantly higher risk of stroke mortality [71]. In a recent United Kingdom study, researchers compared a land use database for green space and compared it to national mortality records from the United Kingdom Office for National Statistics. They found the same independent association between residence in the most green areas with lower rates of dying from circulatory diseases and all cause mortality. Since greater access to green space may simply be a surrogate marker for the other health advantages (healthcare access, nutrition, lower cumulative stress levels, cortisol etc) in affluent 'green' neighborhoods, the researchers controlled for socio-economic status. Green space, it was reported, filled in the gap in health inequalities. Among those with low income and high levels of residential greenery, the mortality rates vs. those with higher socio-economic status were similar. However, when low income was associated with little surrounding green space, the differences in mortality rates became clearly visible. The researchers concluded that green space was an independent variable capable of saving thousands of lives per year in lower income populations [72]. There is, of course, the possibility that much of the epidemiological findings in favor of green space as a variable in health, and mental health in particular, is simply due to green space providing opportunity for physical activity. Given the sound relationship between physical activity and mental health this would be a reasonable presumption. However, there is also evidence indicating that exercise conducted in outdoor settings or green space may be of more value to mental health, physical performance, and motivation to maintain exercise adherence [73-80].

## Nature, urban growth and environmental implications

Within the next several decades the human transition from rural to city residence will accelerate at an even faster rate, with some 90 percent of North Americans and 70 percent of global residents projected to call a city their home [81]. Humans are incredibly social creatures, so it is not at all un-natural that urban centers should grow and thrive. There are, however, some alarming concerns with this inevitable trend. Research shows that cities are far from a panacea for mental health disorders, indeed, rates of depression, anxiety and schizophrenia are consistently reported to be higher among urban residents [33]. Based on the research discussed above, and assuming for a moment that it grows more robust in its scientific strength, the need for access to urban green space may be a mental health necessity. Access to green space and other natural settings affords opportunity for connectivity to nature, and this connectivity, in turn, may provide a layer of insulation against the psychological downsides to urban living; among almost 550 urban men and women, higher scores on the connectivity to nature scale was associated overall psychological well-being, vitality and meaningfulness in life [82]. These strong connections between nature connectivity and personal well-being are found broadly in the population - from private sector executives, high-ranking government employees, to university students, the positive relationship is evident [83]. Urban green space also provides opportunity for contemplation and mindfulness, and a recent study involving over 450 university students shows that mindfulness appears to act as a conduit between connectivity to nature and overall psychological well-being [84].

Some intriguing research suggests that there may be a two-way interaction between the potential mental health benefits of nature and the maintenance of biodiversity. A number of studies have shown that experience in nature, higher connectedness to nature, fosters pro-environmental attitudes and behaviors [85-90]. On the other hand, preliminary investigations have shown that species biodiversity is a variable in the ability of urban green space to influence mental well-being. In a United Kingdom study it was reported that the mental health benefits of 15 different urban green space settings were positively associated with a greater richness of various plant and bird species within these local settings [91]. Australian researchers have extended these results, and even after controlling for various confounders, well-being within urban neighborhoods was associated with species variety and abundance of local birds and totality of vegetation cover [92]. These are important lines of research, particularly when recent evidence suggests that internet-based learning may dilute knowledge and protective concerns related to local biodiversity, in favor of more glamorous species residing in distant locales [93]. There may be a payoff to both personal mental well-being and environmental efforts by raising awareness of potential psychological benefits of local green space and its biodiversity. Canadian researchers have recently reported that contact with nature can foster positive mood state, which in turn facilitates a sense of nature relatedness. The researchers evaluated the psychological effects of walking different routes taken by young adult volunteers - one through buildings and tunnels and the other outdoors through mixed green space - to specific locations in and around the campus. Walking for just 15 minutes through green space, as expected, was associated with more positive post-walk mental outlook. However, the researchers also discovered that the university students were unable to forecast, prior to the walks, that taking differing indoor and outdoor routes could influence mood [94]. A lack of anticipation of benefits derived from urban nature might be cause for alarm, particularly if there is indeed a legitimate displacement of nature-based contact via the omnipresent screen. Although the erosion of our connection to nature may be obscuring its perceived benefits, and research does show that young adults in university settings continue to have minimal awareness of and concern about global climate change and other environmental issues [95], there is reason for optimism - critically, the researchers also showed that walking in nature lifted mood, and mood elevation via nature exposure appears to increase relatedness to nature. The researchers refer to this as a happy path to sustainability, a cycle that can be maintained by fostering awareness that nature has the potential to influence mood [94].

## Future directions

Our review, since it is neither a meta-analysis or systematic review, may unwittingly give the impression that nature's influence in quality of life, stress reduction, mental health and even longevity is positive and iron-clad. However, it must be acknowledged that not all studies have found benefit. For example, a recent cross-sectional study of Japanese adults (average age 52) found no association between the frequency of forest walking and the prevalence of hypertension [96]. Moreover, an examination of 49 of the largest cities in the United States did not find that green space coverage was associated with mortality from heart disease, diabetes, or lung cancer. The US cities with higher green space coverage are more sprawling and associated with greater use of motor vehicles [97]. It is also true that natural settings, forests and wilderness areas in particular, are not without risk. These settings can be the habitat of animals that require their own space, and the risk of contact with vectors carrying infectious diseases (tick-borne diseases as one example) increases in these areas [98-101]. In addition, the Japanese experience with new-growth cedar forests and their association with increasing rates of cedar pollinosis raises questions about the types of trees and plants that are most suitable for parks and urban forests [102-104]. Interestingly, a large prospective study has recently indicated that nature-based occupation (farm work) is associated with reduced risk of developing cedar pollinosis in Japan [105].

In short, there are many questions that researchers must address in the years ahead. Are there individual and cultural differences in preference for natural settings that can influence health outcomes? What might be an appropriate "dose" (duration and frequency) of nature contact? What are the mechanisms of action and what groups of individuals (e.g. children, older adults, and individuals living in deprived communities, those with mental health disorders) might have the most to gain from nature contact [106]? Are certain types of activities (e.g. gardening, walking in forest settings, contemplating in an urban park) more effective than others? How does technology and "virtual nature" fit in? To what extent are human behaviors being dictated by *lack *of nature contact around the home [107]? How does all of this fit into conservation efforts and global environmental issues? Research-based answers to these and other questions should provide helpful insight to policy makers and planners as our global cities expand.

## Conclusion

The available evidence suggests that nature does minister to the mind, and there are more than a few scientific hints suggesting that individuals may need to be made more aware of the potential psychological benefits of nature. A century ago Sir J. Arthur Thomson maintained that the millennia had shaped the far-reaching relations between humans and nature, and that these relations could not be ignored, could not be abandoned, without loss in the realm of positive mental health. While it is difficult to determine to what extent the potential losses might be, it seems fair to suggest that the losses may be more than currently appreciated by most physicians and mental health experts. A lack of anticipated psychological benefits of time spent in nature, as recently reported among a group of young adults on an urban campus, suggests that we have indeed, as Sir J. Arthur Thomson feared, "*put ourselves beyond a very potent vis medicatrix*". Given the positive relationship between nature connectedness, personal well-being, and conservation/pro-environmental attitudes, the experience of even nearby nature might also provide a more sustainable path towards sustainability. Ultimately, an awareness of *vis medicatrix naturae *in the framework of positive psychology can sidestep the dominant negative messaging associated with sustainability and biodiversity. This otherwise fear-based approach is one wherein well-intentioned individuals may be more likely to feel disempowered and throw in the environmental towel [108]. Hopefully, further research will continue to shed light on the ways in which excessive screen time and displacement of time spent in nature might interact to influence mood and cognition. In the meantime, there is enough evidence to suggest that screen time quotas and nature as an opportunity for physical activity, contemplation and mindfulness, are worthy talking points in clinical settings.

## Competing interests

The authors declare that they have no competing interests.

## Authors' contributions

ACL and EMS contributed equal time and effort in the investigation, research and drafting of this manuscript. All authors read and approved the final manuscript.
